# Clot waveform of APTT has abnormal patterns in subjects with COVID-19

**DOI:** 10.1038/s41598-021-84776-8

**Published:** 2021-03-04

**Authors:** Takuya Shimura, Makoto Kurano, Yoshiaki Kanno, Mahoko Ikeda, Koh Okamoto, Daisuke Jubishi, Sohei Harada, Shu Okugawa, Kyoji Moriya, Yutaka Yatomi

**Affiliations:** 1 Department of Clinical Laboratory Medicine, Graduate School of Medicine, The University of Tokyo, 7-3-1 Hongo, Bunkyo-ku, Tokyo, 113-8655 Japan; 2 Department of Infectious Diseases, The University of Tokyo Hospital, Tokyo, Japan; 3 Department of Infection Control and Prevention, The University of Tokyo Hospital, Tokyo, Japan

**Keywords:** Haematological diseases, Infectious diseases

## Abstract

In Coronavirus disease 2019 (COVID-19) subjects, recent evidence suggests the presence of unique coagulation abnormalities. In this study, we performed clot waveform analyses to investigate whether specific modulations are observed in COVID-19 subjects. We analyzed the second derivative of the absorbance in routine APTT tests performed using an ACL-TOP system. We observed high frequencies of abnormal patterns in APTT second-derivative curves that could be classified into an early shoulder type, a late shoulder type, or a biphasic type, high maximum first-derivative and second-derivative peak levels, and a low minimum second-derivative peak level in COVID-19 subjects. These modulations were not observed in subjects with disseminated intravascular coagulation. These abnormal patterns are also observed in patients with lupus anticoagulant, hemophilia, or factor IX deficiency. The plasma fibrinogen levels might also be involved in the abnormal APTT waveforms, especially the high maximum first-derivative and second-derivative peak levels. The abnormal patterns in the APTT second-derivative curves appear with highest frequency at around 2 weeks after the onset of COVID-19 and were not associated with the severity of COVID-19. These results suggest the possible presence of a specific abnormal coagulopathy in COVID-19.

## Introduction

Coronavirus disease 2019 (COVID-19), caused by the 2019 novel coronavirus (SARS-CoV-2), is spreading and threatening populations worldwide. Acute respiratory distress syndrome and multi-organ dysfunction in COVID-19 causes mortality in patients with severe COVID-19. Along with these complications, the activation of coagulation is one of the severe complications of COVID-19. Disseminated intravascular coagulation (DIC), which is commonly associated with sepsis, can be observed in patients with severe COVID-19. However, the pattern of DIC in COVID-19 patients reportedly differs from that in subjects with sepsis. In COVID-19 subjects, an elevation in D-dimer is prominent, while thrombocytopenia and the prolongations of PT and APTT are milder^1–3^. Moreover, a high frequency of venous and arterial thromboembolism is observed, even in subjects with mild COVID-19^4–8^. These unique patterns of laboratory data and the high incidence of thromboembolism suggest that coagulation abnormalities might exist in subjects with COVID-19.

At present, several mechanisms have been proposed to explain the mechanism of abnormal coagulopathy observed in COVID-19 subjects. One proposed mechanism is that SARS-CoV2 infects endothelial cells and is then prone to cause endotheliitis, which results in the acceleration of coagulation^9^. Theoretically, hypo-oxygenation caused by pneumonia can exacerbate endothelial injuries. Actually, the count of circulating endothelial cells was correlated with the severity of COVID-19^10^. Another possible mechanism is the involvement of inflammation. Severe COVID-19 is associated with an elevation of inflammatory cytokines, which can increase the expression of tissue factor and the suppression of anticoagulant factors^11^. A hypofibrinolysis and a high thrombin generation were also reportedly involved in the thrombosis in COVID-19^12^. Recently, lupus anticoagulant has frequently been observed in COVID-19 subjects, and this could also be another possible mechanism of abnormal coagulopathy^13^. The involvement of the increased release of tissue factors^14^, neutrophil extracellular traps^15^, and direct activation of platelets^16^ have also been demonstrated. However, the exact mechanisms responsible for the coagulopathy observed in COVID-19 remain to be elucidated.

In this study, we focused on the clot waveforms of APTT. Clot waveform analyses provide us with information on fibrin abnormalities and the speed and acceleration of clot formation^17^. Several studies have proposed that clot formation curves can suggest some specific diseases^18,19^. In the present study, therefore, we aimed to investigate the clot waveforms of APTT in COVID-19 subjects to investigate whether abnormal patterns of clot formation are observable using these laboratory tests.

## Results

### High frequency of abnormal patterns for second-derivative curves of APTT clot waveforms in COVID-19 subjects

**Figure 1:**
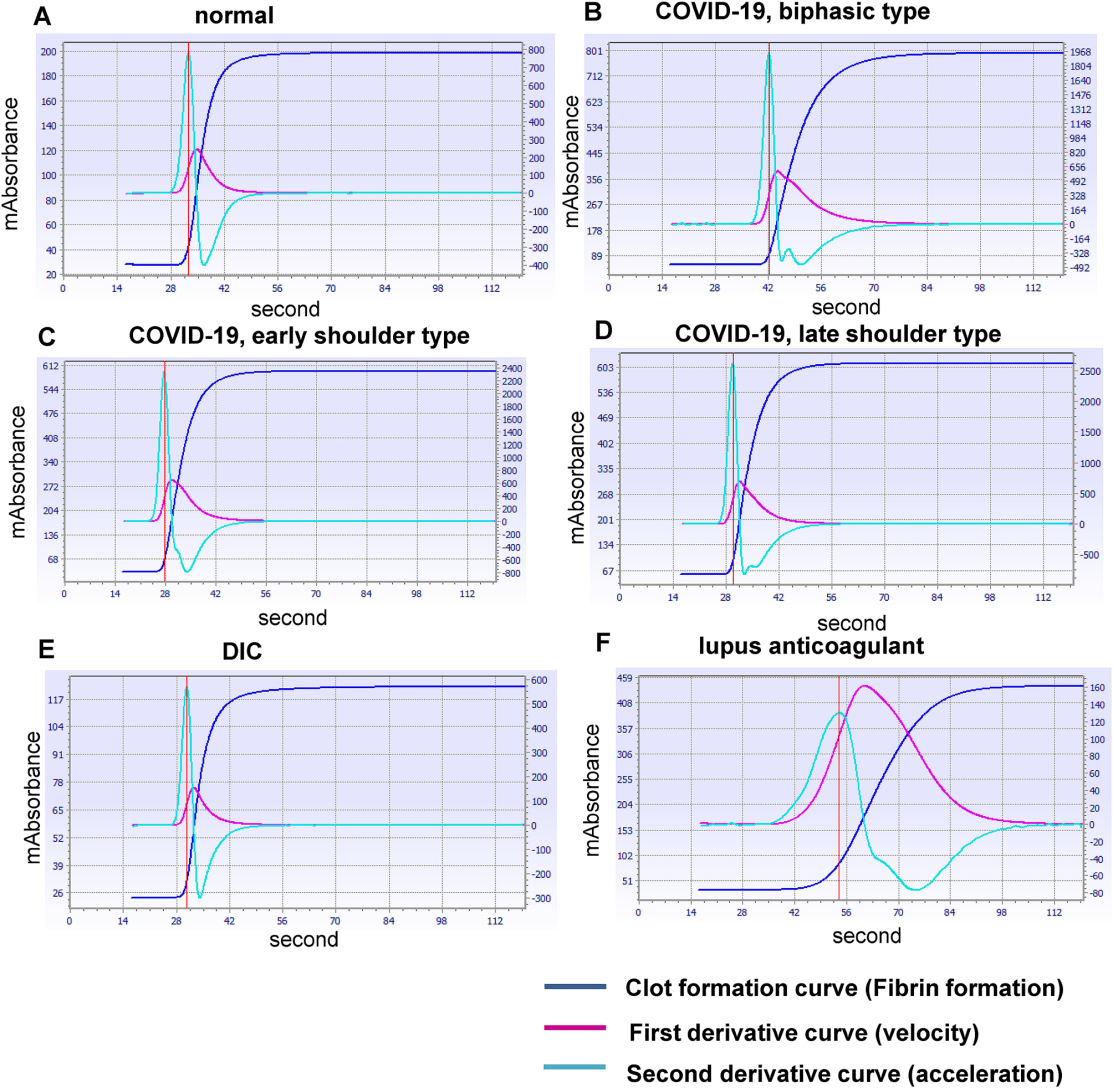
Representative APTT clot waveforms in COVID-19 and other subjects. Representative APTT clot waveforms in COVID-19 and other subjects are shown. (**A**) Normal subject, (**B**–**D**) COVID-19 subjects, (**E**) DIC subject, and (**F**) subject with lupus anti-coagulant.

We found that the second-derivative curves of APTT tests in several COVID-19 subjects showed abnormal patterns: compared with those for normal subjects (Fig. 1A), the abnormal APTT second-derivative curves were classifiable into a biphasic type, an early shoulder type, and a late shoulder type (Fig. 1B–D). In other subjects suffering from other coagulopathies, these abnormal waveform patterns were rarely observed. We did not observe abnormal patterns in subjects with DIC or in those treated with heparin (Fig. 1E and Supplementary Figure S1A). The early shoulder pattern was observed in subjects with lupus anticoagulant (Fig. 1F) and in one subject with FIX deficiency (Supplementary Figure S1B), while the biphasic pattern was observed in one subject with hemophilia and one subject taking warfarin (Supplementary Figure S1C and S1D).

**Figure 2:**
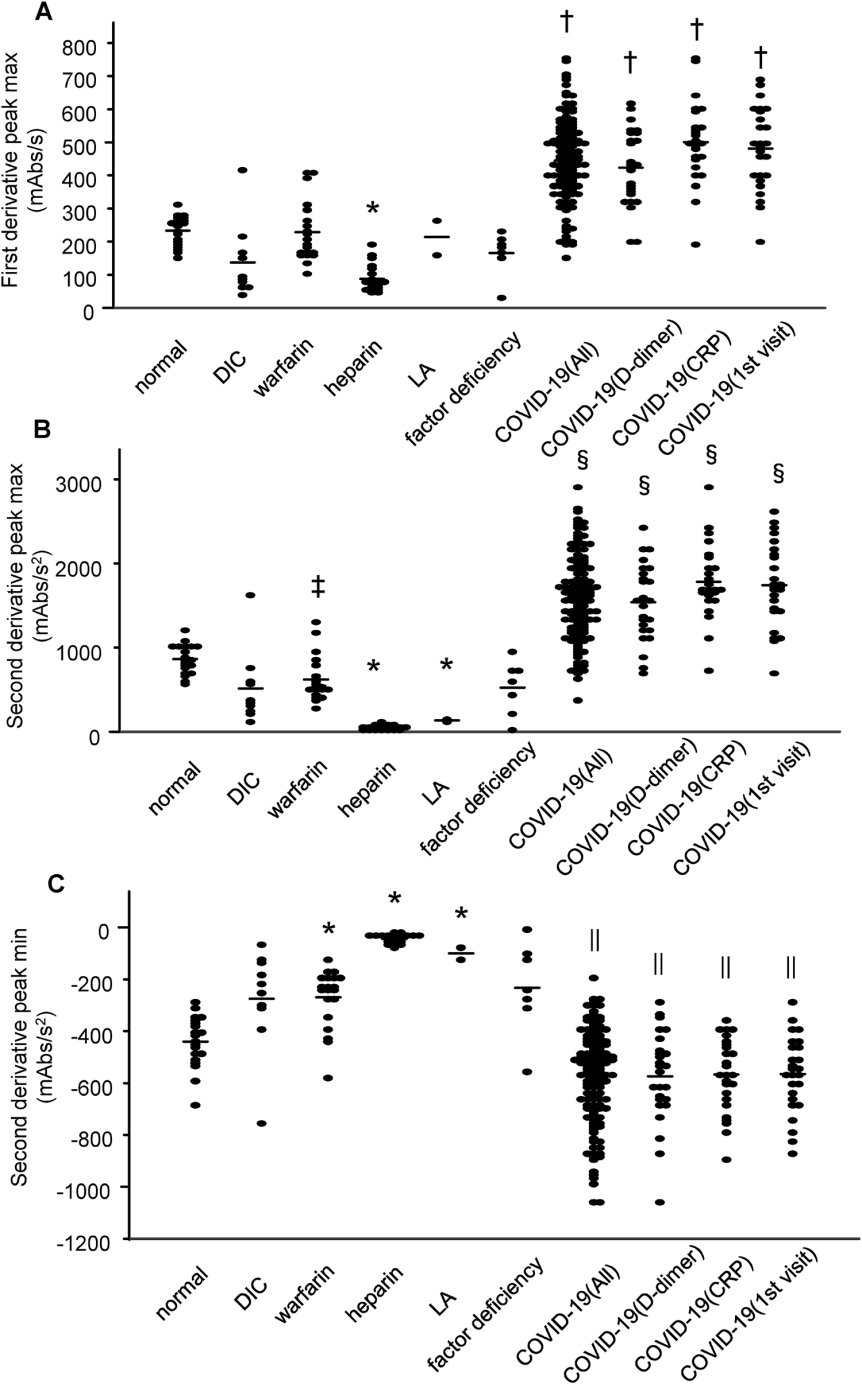
Parameters of APTT derivative curves for COVID-19 subjects. The maximum first-derivative peak (**A**), the maximum second-derivative peak (**B**), and the minimum second-derivative peak (**C**) for COVID-19 subjects are shown. COVID-19 (All), COVID-19 (D-dimer), COVID-19 (CRP), and COVID-19 (1st visit) represent the following data analyses: all APTT tests that were performed, APTT tests performed at time points corresponding to the maximum D-dimer or CRP level during each subject’s time course, and APTT tests performed at the time of each subject’s initial visit to the hospital, respectively. * *P* < 0.01 vs. normal, †*P* < 0.01 vs. other groups except LA, ‡*P* < 0.05 vs. normal, §*P* < 0.01 vs. other groups, ||*P* < 0.05 vs. other groups.

We investigated the frequency of abnormal second-derivative curves by analyzing all the APTT tests together, by analyzing the APTT tests performed when the D-dimer or CRP level was at a maximum during the time course, or by analyzing the APTT tests performed when the subjects visited the hospital for the first time. As shown in Table 1, the frequency of abnormal second-derivative curves was significantly high among the COVID-19 subjects in all the models that were investigated. In addition to the abnormal patterns, the maximums of the first-derivative peak and the second-derivative peak were higher and the minimum of the second-derivative peak was lower among the COVID-19 subjects in all the analyzed models (Fig. 2). Regarding related laboratory data, the APTT, the D-dimer, and the PT levels were significantly higher in the COVID-19 subjects than in the normal subjects, while they were not different between the DIC subjects and the COVID-19 subjects (Supplementary Figure S2A, C, and D). The plasma fibrinogen levels were significantly higher in the COVID-19 subjects than in the DIC subjects (Supplementary Figure S2B).

**Table 1:** Frequency of abnormal patterns of APTT second-derivative curves.

	Normal	Early	Late	Biphasic
Normal	100% (n = 20)	0% (n = 0)	0% (n = 0)	0% (n = 0)
DIC	100% (n = 11)	0% (n = 0)	0% (n = 0)	0% (n = 0)
Warfarin	95% (n = 19)	0% (n = 0)	0% (n = 0)	5% (n = 1)
Heparin	100% (n = 20)	0% (n = 0)	0% (n = 0)	0% (n = 0)
LA	0% (n = 0)	100% (n = 2)	0% (n = 0)	0% (n = 0)
Factor deficiency	72% (n = 5)	14% (n = 1)	0% (n = 0)	14% (n = 1)
COVID-19 (All)	51% (n = 70)	5% (n = 7)	21% (n = 29)	23% (n = 31)
COVID-19 (D-dimer)	58% (n = 15)	4% (n = 1)	15% (n = 4)	23% (n = 6)
COVID-19 (CRP)	27% (n = 7)	4% (n = 1)	31% (n = 8)	38% (n = 10)
COVID-19 (1st visit)	35% (n = 9)	4% (n = 1)	27% (n = 7)	35% (n = 9)

χ^2^ < 0.01 for all the analytical models when all the APTT tests were analyzed together, when only the APTT tests performed at a time point corresponding to the maximum D-dimer or CRP level were analyzed, or when only the APPT tests performed at the time of each subject’s initial visit to the hospital first were analyzed.

### Association between plasma fibrinogen levels and abnormal APTT second-derivative curves in COVID-19 subjects

**Figure 3:**
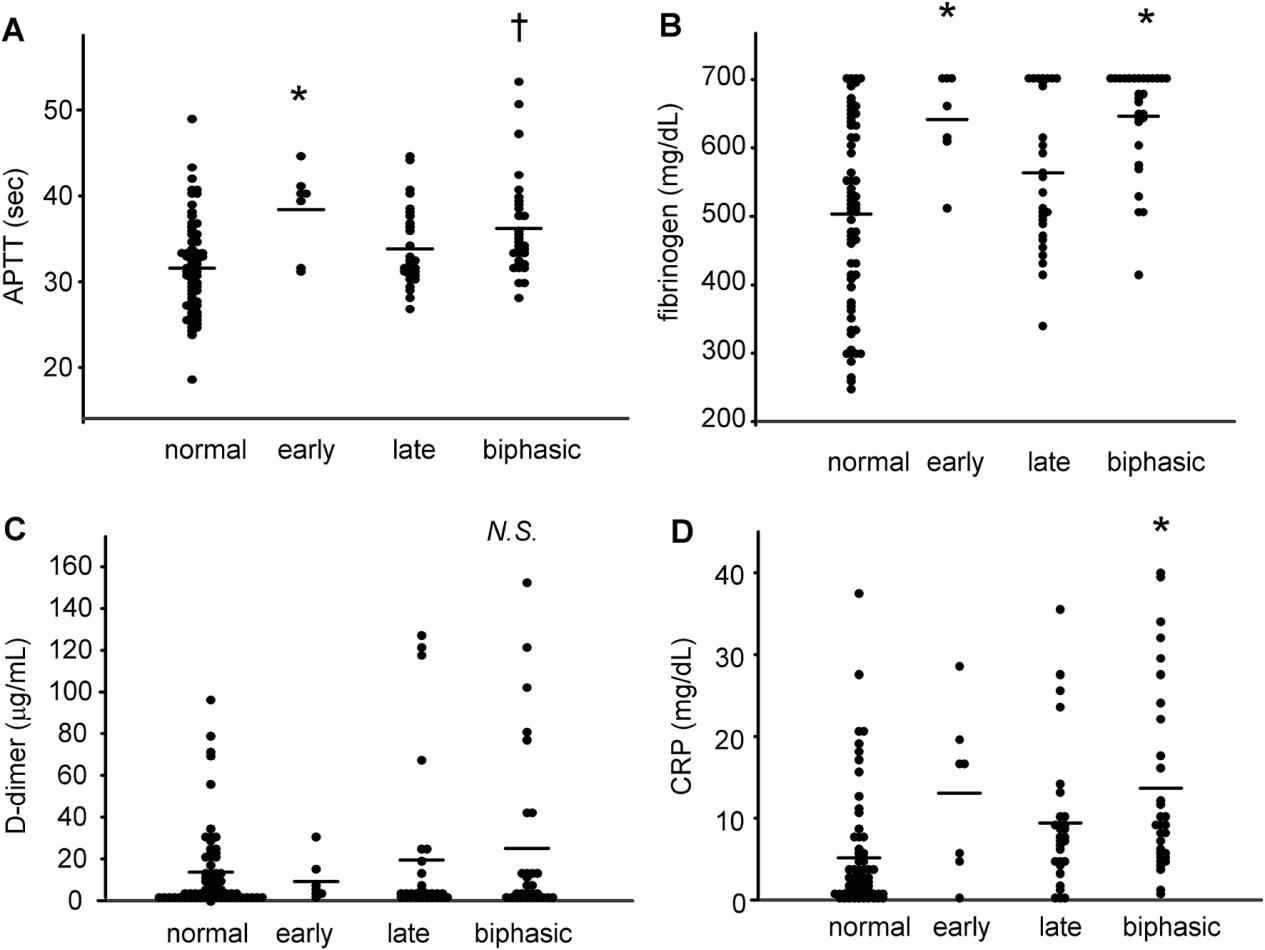
Differences in laboratory data related to coagulation among the patterns of APTT second-derivative curves. (**A**) APTT, (**B**) plasma fibrinogen levels, (**C**) D-dimer levels, and (**D**) CRP levels in specimens with normal, early shoulder, late shoulder, and biphasic patterns of APTT second-derivative curves. * *P* < 0.05 vs. normal, †*P* < 0.01 vs. normal.

Next, we investigated the factors associated with the abnormal APTT second-derivative curves. First, we investigated the potential associations between abnormal APTT second-derivative waveforms and other related factors. We observed that the APTT second-derivative waveforms and the fibrinogen levels were both higher in the specimens with an early shoulder or biphasic pattern and that the CRP levels were higher in the specimens with a biphasic pattern, compared with those with a normal pattern, while the D-dimer levels and the platelet counts did not differ according to the waveform pattern (Fig. 3 and Supplementary Figure S3D). The levels of PT, AST, ALT, and total bilirubin were not different among the types of clot waveforms (Supplementary Figure S3E–H). The maximum first-derivative peak and the maximum second-derivative peak were significantly higher in the specimens with a biphasic pattern (Supplementary Figure S3A–C).

**Figure 4:**
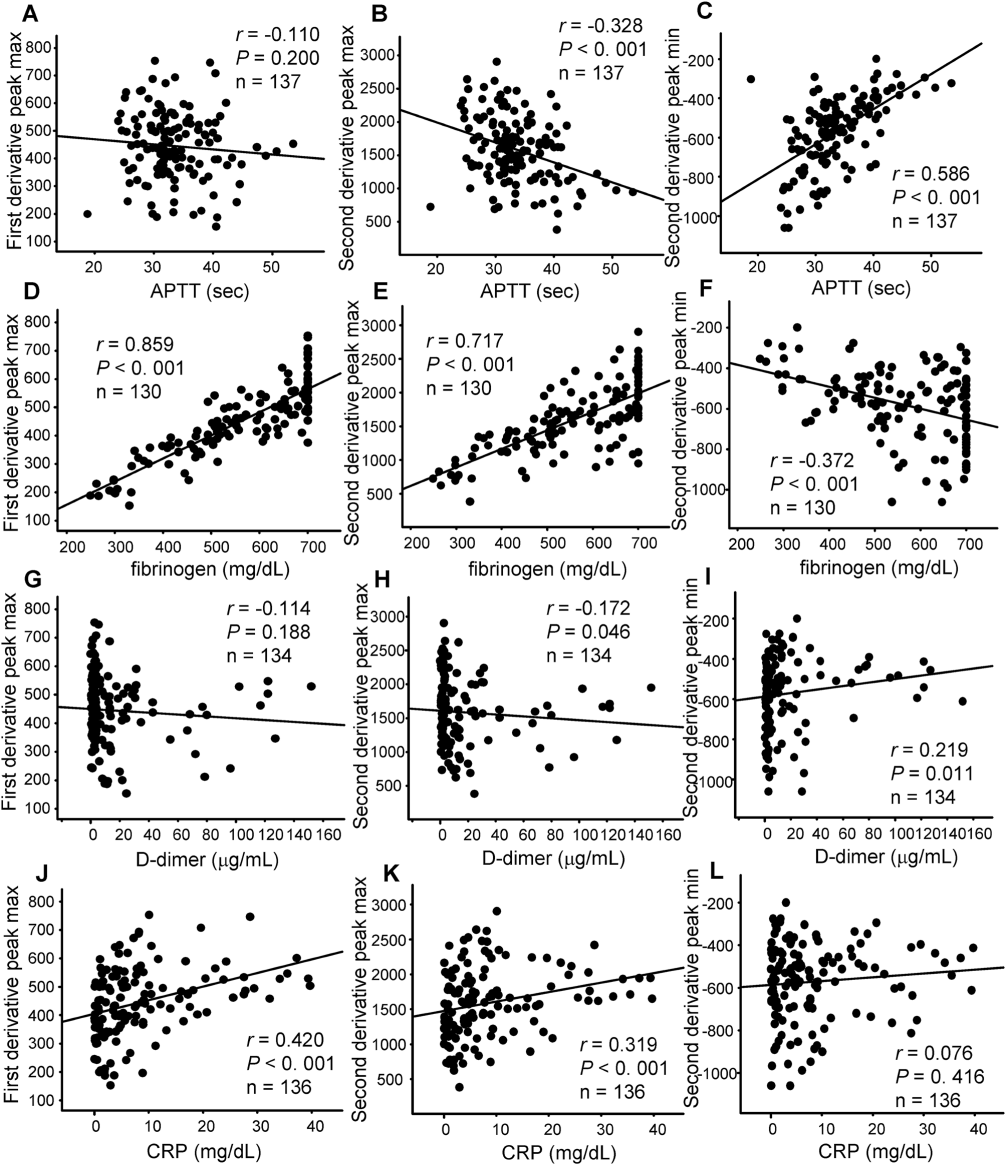
Correlations between parameters of APTT derivative curves and laboratory data related to coagulation in COVID-19 subjects. Correlations between the maximum first-derivative peak (**A**, **D**, **G**, **J**), the maximum second-derivative peak (**B**, **E**, **H**, **K**), and the minimum second-derivative peak (**C**, **F**, **I**, **L**) and APTT (**A**–**C**), fibrinogen (**D**–**F**), D-dimer (**G**–**I**), and CRP (**J**–**L**).

Regarding the association between the maximum first-derivative peak or the maximum or minimum second-derivative peak and other laboratory data, we found rather strong associations between the maximum first-derivative peak or the maximum second-derivative peak and the plasma fibrinogen levels and a moderate association between the minimum second-derivative peak and the APTT level (Fig. 4 and Supplementary Figure S4). Although abnormal APTT waveforms were also observed in subjects without elevated fibrinogen levels, these results suggest that the appearance of abnormal APTT waveforms in COVID-19 subjects might be associated with pathological situations involving an increase in fibrinogen.

### Appearance of abnormal patterns in APTT second-derivative curves with highest frequency at around 2 weeks after the onset of COVID-19

**Figure 5:**
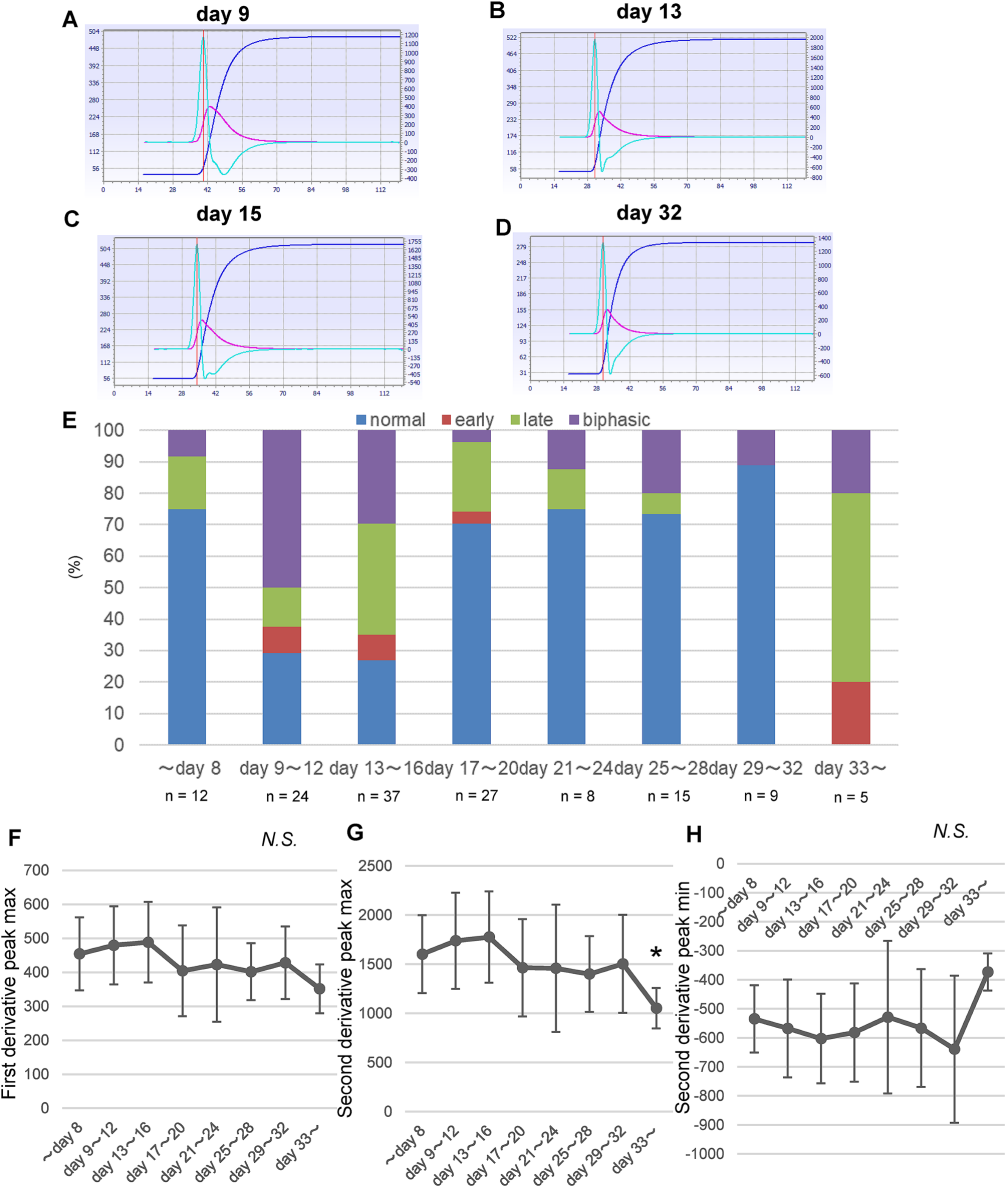
Time course of the frequency of abnormal patterns and parameters of APTT derivative curves among COVID-19 subjects. (**A**–**D**) Time course of APTT clot waveforms in one COVID-19 subject. (E–H) Time course of the frequency of abnormal patterns and parameters of APTT derivative curves among COVID-19 subjects. * *P* < 0.05 vs. ≤ day 8, *P* < 0.01 vs. days 9–12 and days 13–16.

Next, we investigated the time course of the APTT second-derivative curves of COVID-19 subjects. Figure 5A–D shows the time course of the APTT second-derivative curves for a COVID-19 subject who suffered from severe COVID-19 followed by septic DIC after the disappearance of SARS-CoV2. The time course for the APTT second-derivative curves supports the idea that the appearance of a biphasic pattern might be rather specific to COVID-19.

Figure 5E–H shows the time course for the incidence of an abnormal pattern in APTT second-derivative curves and the maximum first-derivative peak or the maximum or minimum second-derivative peak. Considering that the data points were limited to tests performed at more than 33 days after the onset of COVID-19, the appearance of an abnormal pattern was highest at around 2 weeks after the estimated onset of COVID-19, while the maximum first-derivative peak and the maximum and minimum second-derivative peaks did not show any obvious changes over time.

### Lack of association between abnormal patterns in APTT second-derivative curves and the severity of COVID-19

Lastly, we investigated whether the existence of a biphasic pattern for the APTT second-derivative curve was associated with any of the clinical characteristics that were examined in this study. As shown in Table 2, no association between the severity of the subjects and the existence of an abnormal APTT second-derivative waveform was seen. We did not observe any significance differences in the appearance of an abnormal APTT pattern between mild to moderate cases and severe cases, at the time point when the D-dimer or CRP level reached a maximum during the time course, or at the time of the subjects’ initial hospital visit. We also did not find any significance difference in the maximum first-derivative peak or the maximum or minimum second-derivative peak according to severity (Supplementary Figure S5).

**Table 2:** Differences in the frequency of abnormal APTT second-derivative waveforms according to the severity of COVID-19 subjects.

	Normal	Early	Late	Biphasic
Mild case	62% (n = 18)	7% (n = 2)	17% (n = 5)	14% (n = 4)
Moderate case	48% (n = 30)	0% (n = 0)	24% (n = 15)	27% (n = 17)
Severe case	48% (n = 22)	11% (n = 5)	20% (n = 9)	22% (n = 10)

χ^2^ < 0.01.

Regarding the association with the thrombotic events, three subjects suffered from deep venous thrombosis in the present study. Although we cannot demonstrate statistically since the number of the subjects with the events was small, abnormal patterns of APTT clot waveform were observed in all of the three subjects. On the contrary, the maximum first-derivative peak tended to be lower, the maximum second-derivative peak was significantly lower, and the minimum second-derivative peak was significantly higher in the subjects with deep venous thrombosis than those without it (Supplementary Figure S6), which might reflect that all of the three subjects with deep venous thrombosis had been administered with heparin intravenously.

## Discussion

Abnormal coagulation is emerging as a severe complication in COVID-19 subjects. In addition to a condition resembling DIC, a high incidence of thromboembolism can lead to impairments in COVID-19 patients. In the present study, we analyzed the clot waveforms for APTT tests and found a high frequency of abnormal patterns in APTT second-derivative curves, high maximum first-derivative and second-derivative peak levels, and a low minimum second-derivative peak level in COVID-19 subjects.

Regarding the abnormal APTT second-derivative waveforms, these patterns are sometimes observed in specific deficiencies or inhibitions of coagulation factors, such as hemophilia, or the presence of lupus anticoagulant^20,21^. Concordantly with previous reports, we observed the presence of biphasic patterns in patients with lupus anticoagulant, hemophilia, or factor IX deficiency and in one subject who was taking warfarin (Table 1). Regarding DIC, contrary to a previous report using other coagulation parameters^22^,we did not observe any abnormal patterns in the subjects with DIC. Another group using the same parameters as we have reported similar results^23^. The presence of lupus anticoagulant has been reported in COVID-19 subjects^13^. Although the prolongation of APTT was not observed in the subjects with abnormal clot waveform patterns, we cannot rule out the possibility of its involvement, considering the high incidence of lupus anticoagulant in COVID-19 subjects^13^ and the fact that the prolongation of APTT is not always observed in the subjects with lupus anticoagulant. One possible mechanism is the presence of an inhibitor to specific coagulation factors, possibly explaining the appearance of a biphasic pattern in APTT second-derivative curves^20^. However, the appearance of a biphasic pattern was not associated with the severity of COVID-19 (Table 2), which is not concordant with a possible mechanism in which the consumption of specific coagulation factors results in the abnormal APTT second-derivative waveforms. Another possible mechanism is an abnormality in von Willebrand Factor (VWF), although we did not observe an abnormal waveform in the single subject with von Willebrand disease who was examined in the present study. Tokunaga et al. reported that the plasma level of VWF directly influences the abnormal biphasic patterns of APTT second-derivative curves^19^. VWF is produced and stored in endothelial cells and it increases during thrombogenicity, forming bridges between sub-endothelial collagen and platelets^24^, while it protects FVIII against proteolytic inactivation by activated protein C and S^25^. Since endothelial inflammation and injuries are often observed in COVID-19^9^, an impairment in VWF function because of an abnormal quality, abnormal local circumstance, or abnormal quantity of VWF might occur in COVID-19. At present, several studies have reported that plasma VWF levels, together with FVIII, were higher in COVID-19 subjects^26–28^. The elongation of the APTT and high fibrinogen and CRP levels might be associated with the presence of an abnormal APTT second-derivative waveform; however, specimens with normal APTT, fibrinogen, or CRP values sometimes exhibit abnormal APTT second-derivative waveforms, suggesting that these phenomena or molecules are not the direct causes of the abnormal waveforms.

Regarding the time course of the APTT clot waveforms, as shown in Fig. 5E, the appearance of an abnormal pattern was highest at around 2 weeks. Compared with the previous studies showing the time course and prevalence of abnormal hemostasis parameters, the appearance of an abnormal waveform pattern might be prolonged for longer durations than the elevated levels of D-dimer and PAI-1^29^ and be observed more frequently than the elevation of D-dimer, although the detail information on the durations from the onset of COVID-19 was not available and the types of the subjects and the countries where researches were performed were different^30,31^. These potential differences between the APTT clot waveform and other hemostasis parameters might suggest the possibility that the abnormal APTT clot waveform might reflect some unique abnormalities in coagulation, which might not directly result in the abnormal routine hemostasis parameters.

We also observed high maximum first-derivative and second-derivative peak levels and a low minimum second-derivative peak level in COVID-19 subjects. These results suggest that the speed and acceleration of clot formation were relatively high in APTT tests performed using specimens from COVID-19 subjects. Considering that these parameters are strongly correlated with plasma fibrinogen levels (Fig. 4) and that fibrinogen theoretically enhances the speed and acceleration of clot formation, high fibrinogen levels could explain these modulations in COVID-19 subjects. Fan, B.E.,et al. demonstrated that Factor VIII levels and VWF levels increased together with the raised APTT clot waveform parameters and fibrinogen^27^. As shown in Supplementary Figure S1, we observed the existence of abnormal clot waveform in the subjects with hemophilia, suggesting the possible involvement of Factor IX as well as Factor VIII in the abnormal clot waveform. Although we have not measured them, these factors might be also involved in the abnormal APTT clot waveform. Another possibility is the involvement of several phospholipids or factors that accelerate the response to phospholipids in APTT assays, since an APTT assay monitors clot formation arising from a response to phospholipids. Actually, HCoV-229E, another type of coronavirus, reportedly disturbs phospholipid homeostasis in infected cells^32^. Regarding the clot waveform analyses and COVID-19, recent reports have demonstrated that the first-derivative and second-derivative peak levels were higher, and the minimum second-derivative peak level were lower or tended to be lower in critically ill COVID-19 subjects^27,33,34^. Although in the present study, we observed no difference in the APTT clot waveform parameters among mild, moderate, and severe COVID-19 subjects (Supplemental Figure S5), the modulation of these parameters in the COVID-19 subjects would be concordant with the previous studies, considering the time course of these parameters (Fig. 5F–H) and the difference between the COVID-19 subjects and control subjects (Fig. 2). Further prospective and larger studies are necessary to elucidate the clinical significance of the modulation of these parameters in the COVID-19 subjects.

The limitation of the present study is that the coagulation assays including APTT assays could be affected by many interferences and somehow be dependent on the types of reagents used as described in the previous article^35^. The detail blood oxygen conditions might also affect the results, which were not available in this study. However, considering that we observe no difference in the presence of abnormal clot waveform between mild COVID-19 subjects who did not require oxygen therapy and severe COVID-19 subjects who required mechanical ventilation therapy, we believe that the oxygen values might not affect the abnormal clot waveform in APTT. Moreover, this study examined APTT clot waveforms retrospectively and the timing of the APTT assays was various among the subjects, we could not elucidate the exact underlying mechanism responsible for the abnormal APTT second-derivative curves observed in COVID-19 subjects. We should also admit the number of the subjects was too small in the present study and other markers of haemostasis have not been measured.

In spite of these limitations, the results of this study suggest the possible presence of a specific abnormal coagulopathy in COVID-19. Although further studies are necessary, the fact that abnormal patterns of APTT clot waveform were shown in all of the three COVID-19 subjects with deep venous thrombosis suggested the possible usefulness of APTT clot waveform analysis in evaluating the risk of thrombotic events in COVID-19 subjects. We should also think the possibility of COVID-19 in case when we observe an abnormal APTT clot waveform in the subjects with abnormal coagulopathy and we expect that the present study will help the researchers investigate the pathogenesis of the COVID-19 associated thrombosis.

## Methods

### Subjects

We analyzed the clot waveforms observed for 137 routine APTT tests performed using specimens from 26 COVID-19 subjects confirmed with RT-PCR using primers specific for SARS-CoV-2s. We also performed APTT clot waveform analyses using specimens from 20 normal subjects, 2 subjects with lupus anticoagulant, 10 DIC patients with sepsis, 20 subjects taking warfarin, 20 subjects treated with heparin, and 7 subjects with a congenital absence of specific coagulation factors (FVII deficiency, n = 1; hemophilia B homo-type, n = 1; hemophilia A hetero-type, n = 1; hemophilia A homo-type, n = 3; von Willebrand disease, n = 1). COVID-19 subjects were categorized into three groups: those requiring no oxygen therapy (“mild” group), those requiring oxygen treatment without mechanical respiratory ventilation support (“moderate” group), and those requiring mechanical respiratory ventilation support (“severe” group). The characteristics of the COVID-19 subjects were described in Table 3. Seven subjects were intravenously administered with heparin and two subjects were subcutaneously administered with heparin. Three of 26 subjects suffered from deep venous thrombosis. The current study was performed in accordance with the ethical guidelines of the Declaration of Helsinki. Participants were informed about the study on the website. Patients who rejected to be enrolled in our study were excluded. The study design was approved by The University of Tokyo Medical Research Center Ethics Committee, which waived written informed consent because data in this retrospective study were retrieved from medical records (approval number, 3683).

**Table 3:** Characteristics of the COVID-19 subjects.

Age (mean ± S.D)	63.7 ± 12.8
Sex (male/female, n)	21/5
Maximum severity of COVID-19 (n)	Mild: 3; moderate: 14; severe: 9
Presence of diabetes (%)	23.1
Presence of hypertension (%)	42.3
Presence of current smoking (%)	11.6
Use of favipiravir (%)	76.9
Use of nafamostat (%)	61.5
Use of heparin (%)	34.6

### Clot waveform analyses

The APTT tests were performed using the ACL APTT SyntheSis kit and the ACL-TOP 700 hemostasis testing system (Instrumentation Laboratory, Bedford, Massachusetts, USA), which provides three types of curves: a curve showing the changes in absorbance observed during the APTT measurement; a curve presenting the first derivative of the absorbance, corresponding to the coagulation velocity; and a curve showing the second derivative of the absorbance, corresponding to the coagulation acceleration^19^. We investigated the presence of a biphasic pattern in the clot waveform for the second derivative of the absorbance as well as the parameters calculated from the clot waveform, the maximum peak of the first derivative, and the maximum and minimum peaks of the second derivative. Although the time between the sampling and the performance of the assay was not strictly the same, we performed the APTT assays at least in a few hours after the sampling.

### Measurement of CRP, D-dimer, and fibrinogen

CRP and D-dimer levels were determined by the latex agglutination immunoassay test (LZ test 'Eiken' CRP-HG; Eiken Kagaku Company Limited, Tokyo, Japan and LPIA-ACE; Mitsubishi Chemical Medience Co, Tokyo, Japan). Fibrinogen levels were measured by HemosIL fibrinogen C(II) (Instrumentation Laboratory, Bedford, Massachusetts, USA).

### Statistical analyses

All the data were analyzed statistically using SPSS (Chicago, IL). The results were expressed as dot plots or the mean ± S.D. The differences in the existence of an abnormal clot waveform were assessed using a Chi square test. Values for more than three groups were compared using the Kruskal–Wallis test followed by the Games Howell test as a post-hoc test. Correlations were examined using the Spearman correlation test. *P* values less than 0.05 were regarded as statistically significant in all the analyses.

## Supplementary Information

Supplementary Information.

## Data Availability

The datasets generated or analyzed in the current study are available upon reasonable request.
