# Supplementary material for: Clot waveform of APTT has abnormal patterns in subjects with COVID-19
Source: Sci Rep. 2021 Mar 4;11:5190. doi: 10.1038/s41598-021-84776-8 (PMC7933409; doi:10.1038/s41598-021-84776-8)
Supplement: Supplementary file 1 — Supplementary Information. [file 41598_2021_84776_MOESM1_ESM.pdf]

Clot Waveform of APTT Has Abnormal Patterns in Subjects with COVID-19

Takuya Shimura[1], Makoto Kurano[1*], Yoshiaki Kanno[2], Mahoko Ikeda[2,3], Koh Okamoto[2], Daisuke Jubishi[2], Sohei Harada[3], Shu Okugawa[2], Kyoji Moriya[2, 3], Yutaka Yatomi[1]

[1] Department of Clinical Laboratory, The University of Tokyo Hospital, Tokyo, Japan

[2] Department of Infectious Diseases, The University of Tokyo Hospital, Tokyo, Japan

[3] Department of Infection Control and Prevention, The University of Tokyo, Japan

* Corresponding author

Running Title: Abnormal clot waveform in COVID-19

Address correspondence to:

Makoto Kurano, MD, PhD

Department of Clinical Laboratory Medicine, Graduate School of Medicine,

The University of Tokyo, 7-3-1 Hongo, Bunkyo-ku, Tokyo 113-8655, Japan

Tel: +81-3-3815-5411;

Fax: +81-3-5689-0495;

e-mail: kurano-tky@umin.ac.jp (M.K.)

Supplementary Figure S1

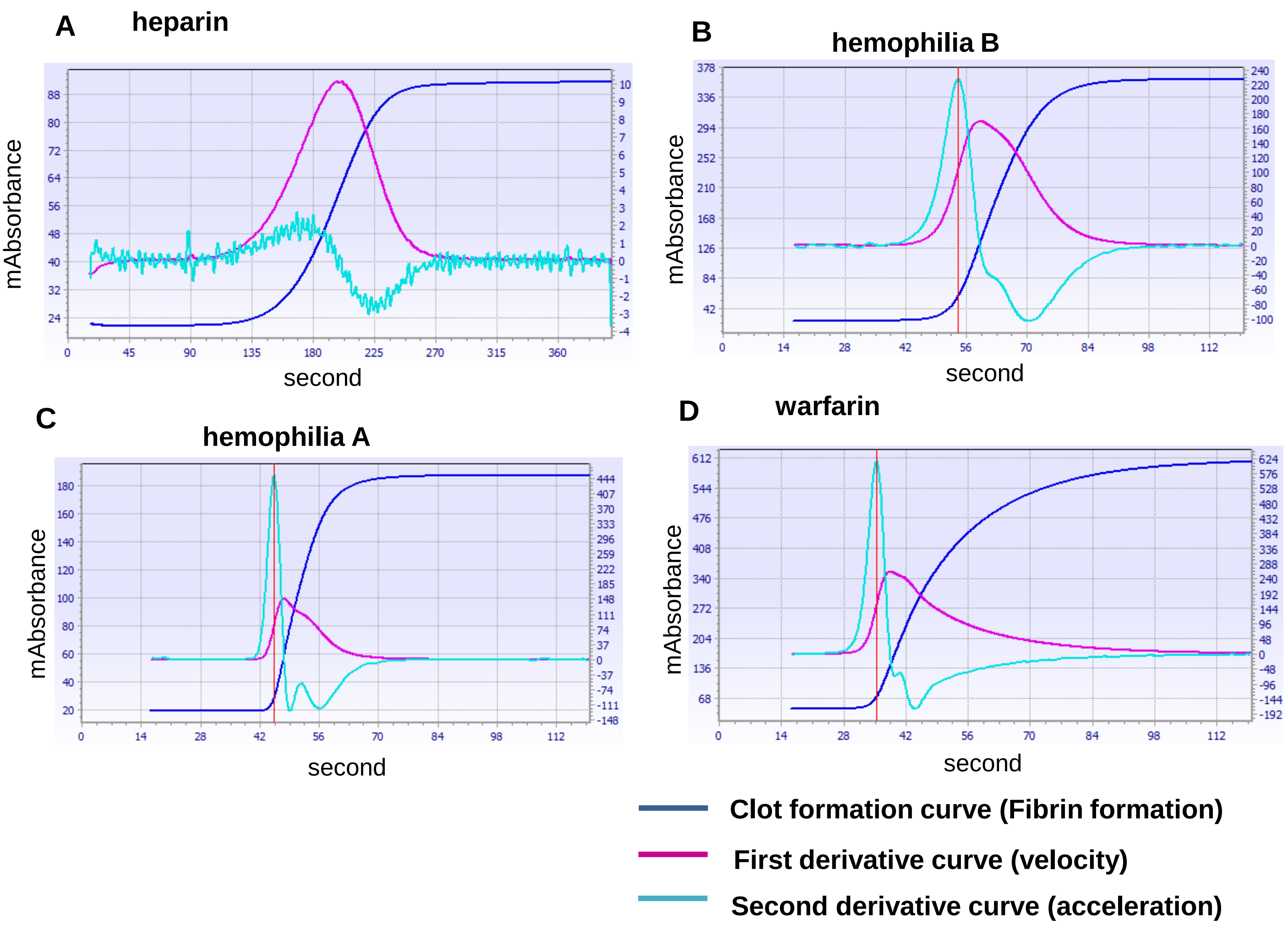

Supplementary Figure S1. Representative APTT clot waveforms in subjects with hemophilia or subjects treated with heparin or warfarin.

(A) Subject treated with heparin, (B) subject with hemophilia B, (C) subject with hemophilia A, and (D) subject treated with warfarin.

Supplementary Figure S2

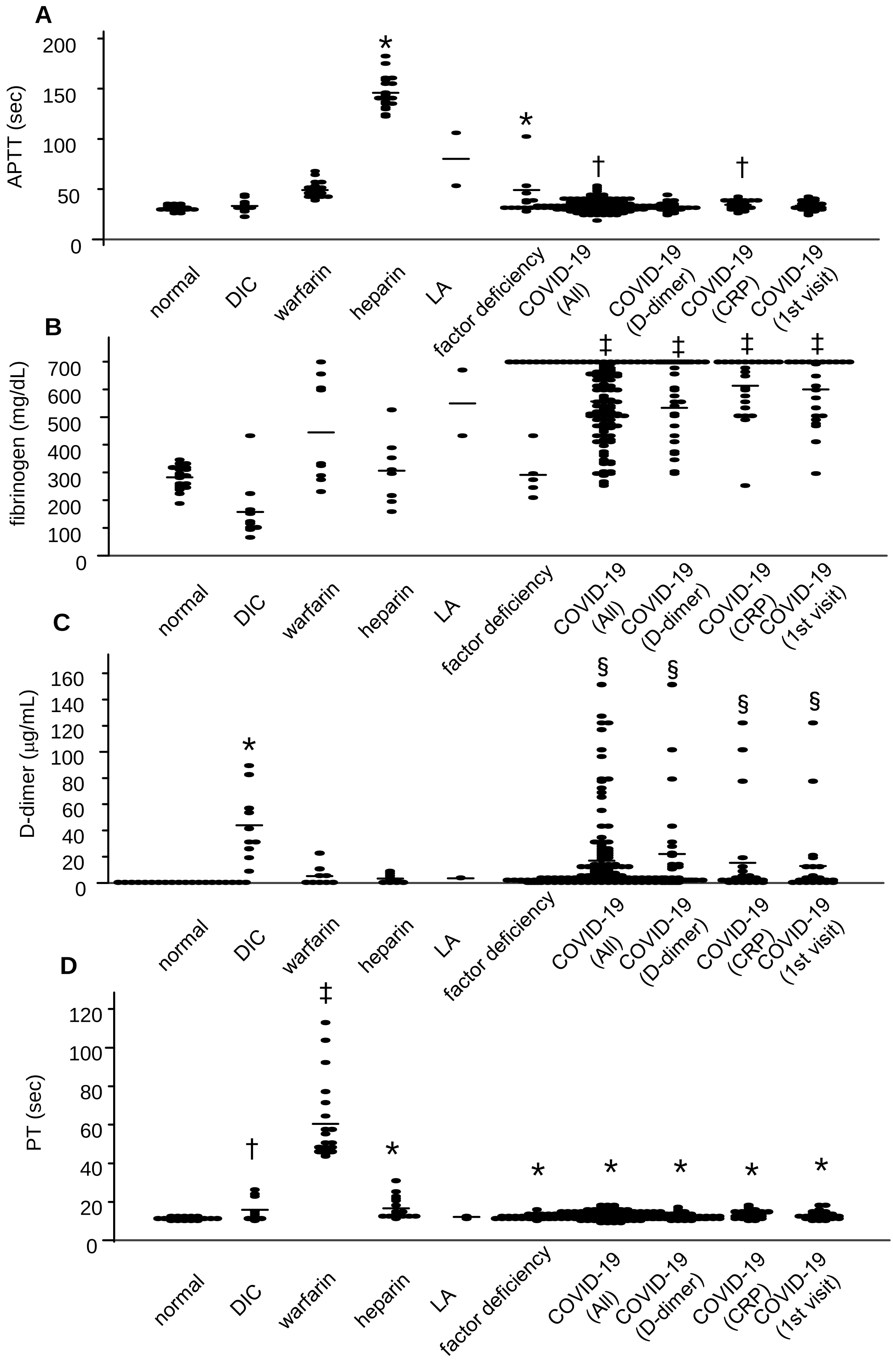

Supplementary Figure S2 (continued)

Supplementary Figure S2. Laboratory data related to coagulation in COVID-19 subjects.
APTT (A), fibrinogen (B), D-dimer (C), and PT levels (D) in COVID-19 subjects. COVID-19 (All), COVID-19 (D-dimer), COVID-19 (CRP), and COVID-19 (1st visit) represent the following data analyses: all APTT tests that were performed, APTT tests performed at time points corresponding to the maximum D-dimer or CRP level during each subject's time course, and APTT tests performed at the time of each subject's initial visit to the hospital, respectively. * $P < 0.01$ vs. normal, †$P < 0.05$ vs. normal, ‡$P < 0.05$ vs. other groups except warfarin and LA, § $P < 0.01$ vs. other groups except DIC.

Supplementary Figure S3

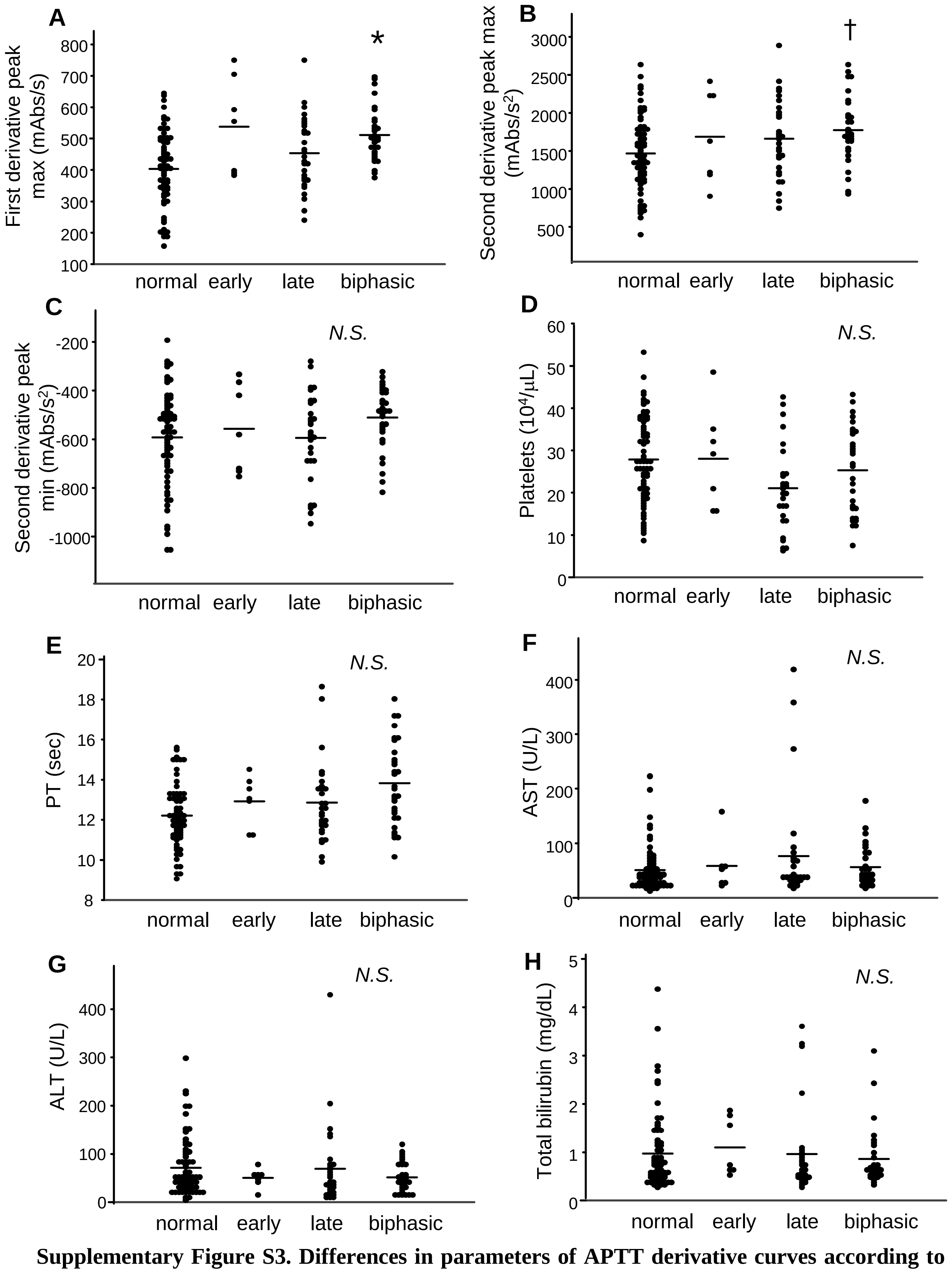

Supplementary Figure S3. Differences in parameters of APTT derivative curves according to patterns of APTT second-derivative curves.

The maximum first-derivative peak (A), the maximum second-derivative peak (B), the minimum second-derivative peak (C), the platelet counts (D), the PT levels (E), the AST levels (F), the ALT levels (G), and the total bilirubin levels (H) for specimens with normal, early shoulder, late shoulder, and biphasic patterns of APTT second-derivative curves. * $P < 0.01$ vs. normal, †$P < 0.05$ vs. normal.

Supplementary Figure S4

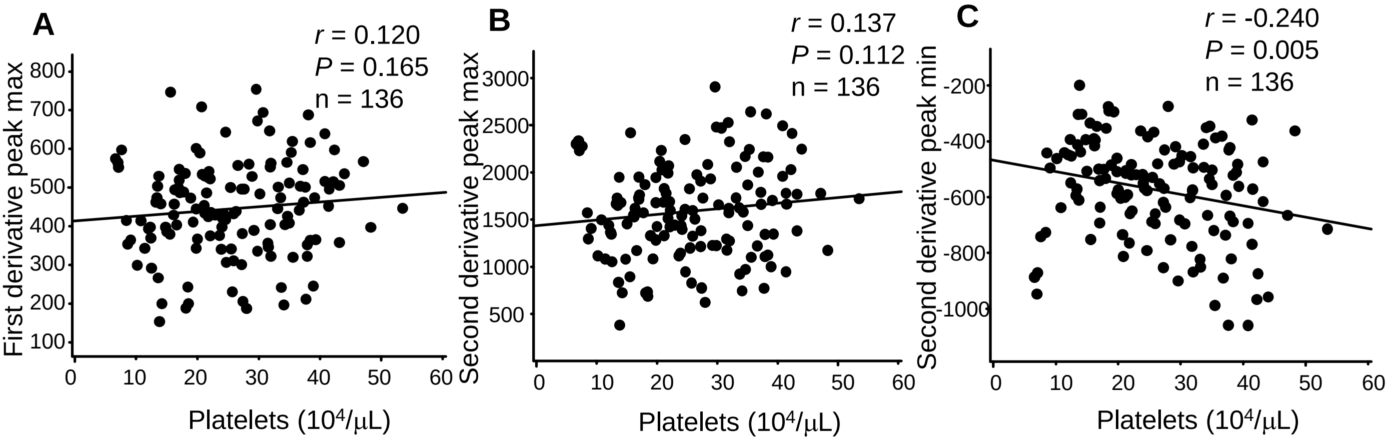

Supplementary Figure S4. Correlations between parameters of APTT derivative curves and platelet counts in COVID-19 subjects.

Correlations between the maximum first-derivative peak (A), the maximum second-derivative peak (B), and the minimum second-derivative peak (C) and the platelet count are shown.

Supplementary Figure S5

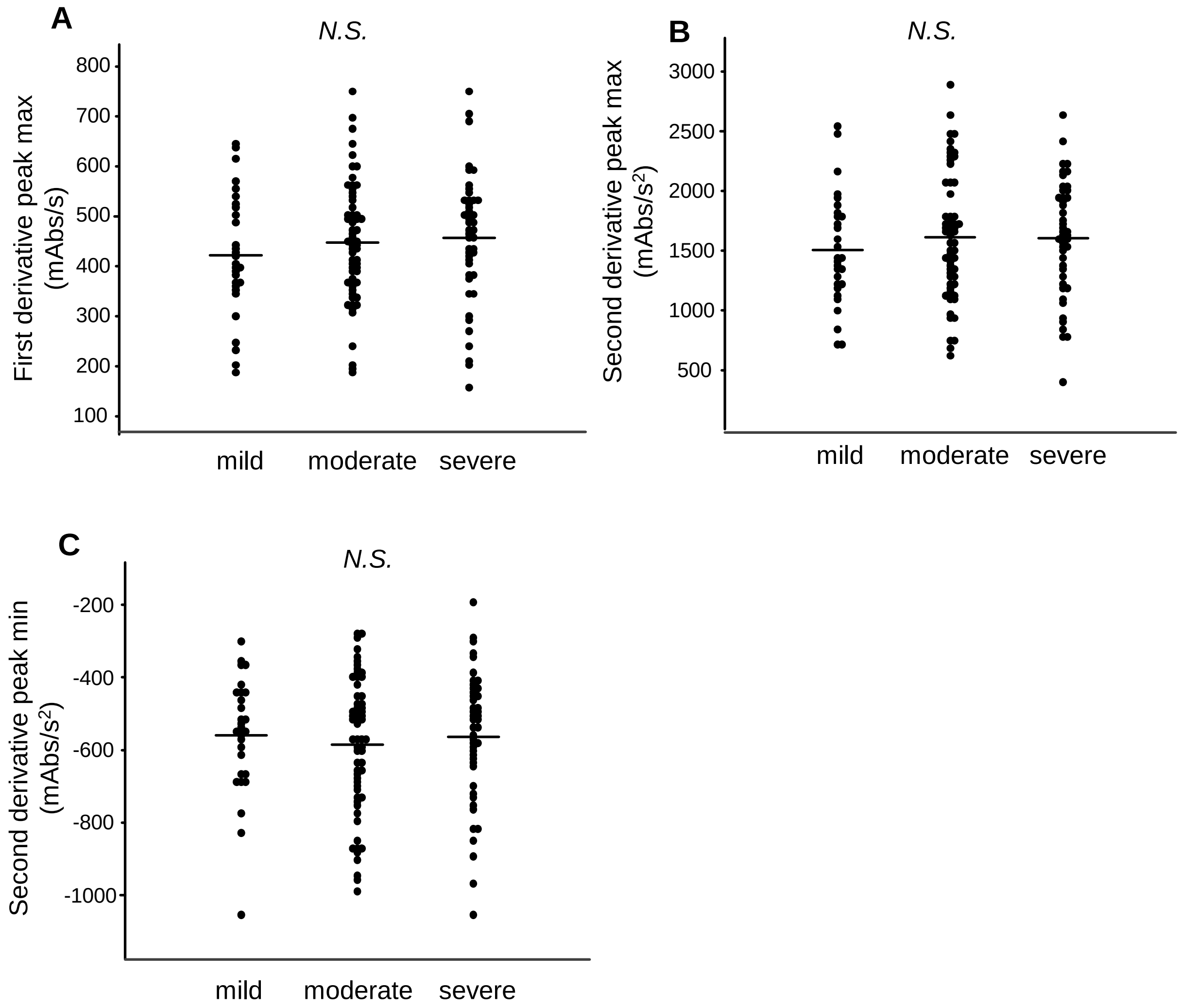

Supplementary Figure S5. Differences in parameters of APTT derivative curves according to the severity of COVID-19.

The maximum first-derivative peak (A), the maximum second-derivative peak (B), and the minimum second-derivative peak (C) according to the severity of COVID-19.

Supplementary Figure S6

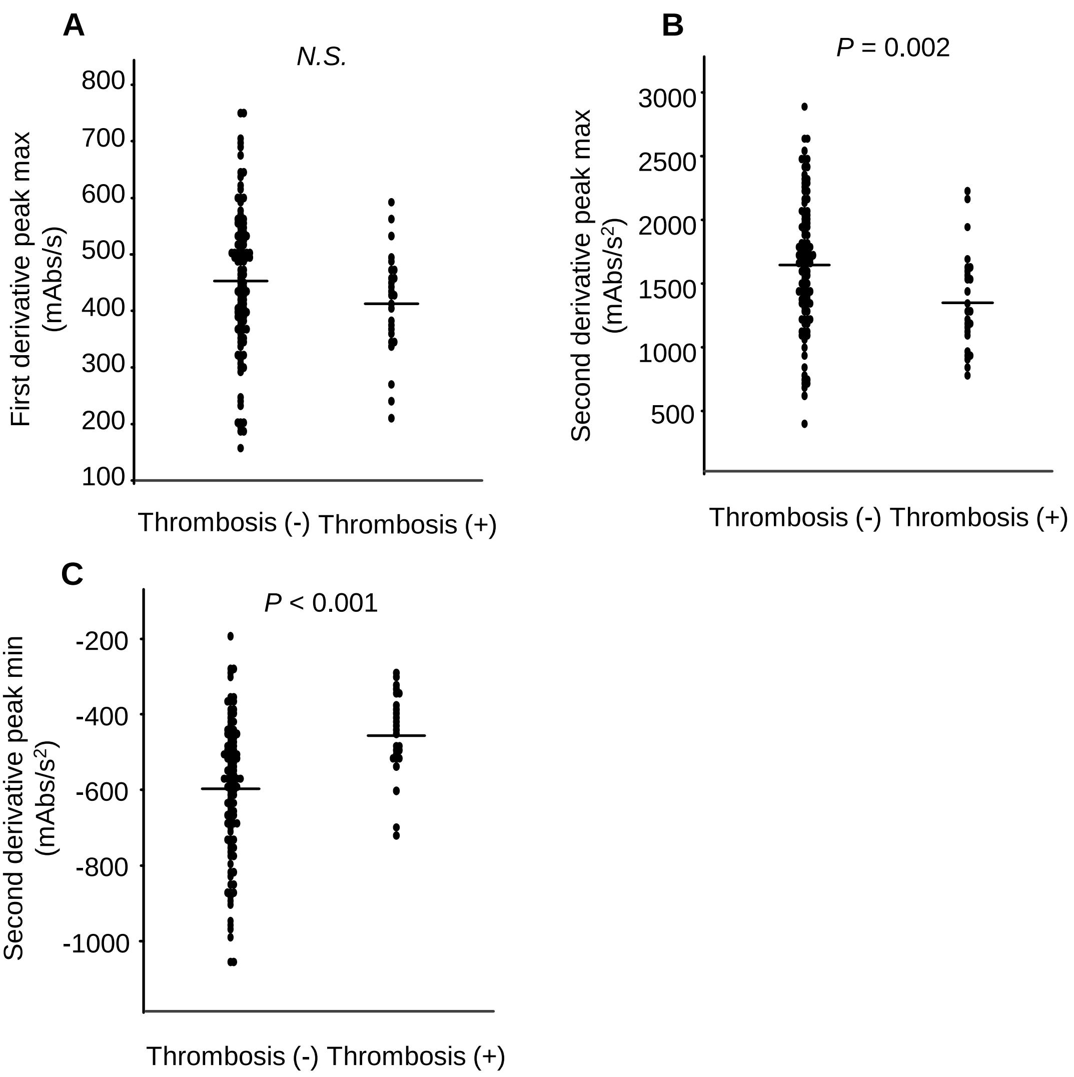

Supplementary Figure S6. Differences in parameters of APTT derivative curves according to the severity of COVID-19.

The maximum first-derivative peak (A), the maximum second-derivative peak (B), and the minimum second-derivative peak (C) according to the incidence of thrombotic events.
